# Differential Water Deficit in Leaves Is a Principal Factor Modifying Barley Response to Drought Stress

**DOI:** 10.3390/ijms232315240

**Published:** 2022-12-03

**Authors:** Małgorzata Nykiel, Marta Gietler, Justyna Fidler, Jakub Graska, Anna Rybarczyk-Płońska, Beata Prabucka, Ewa Muszyńska, Jan Bocianowski, Mateusz Labudda

**Affiliations:** 1 Department of Biochemistry and Microbiology, Institute of Biology, Warsaw University of Life Sciences-SGGW, Nowoursynowska 159, 02-776 Warsaw, Poland; 2 Department of Botany, Institute of Biology, Warsaw University of Life Sciences-SGGW, Nowoursynowska 159, 02-776 Warsaw, Poland; 3 Department of Mathematical and Statistical Methods, Poznań University of Life Sciences, Wojska Polskiego 28, 60-637 Poznań, Poland

**Keywords:** barley, drought, nitrate reductase, L-cysteine desulfhydrase, lipid peroxidation, alert phase

## Abstract

In response to environmental stress, plants activate complex signalling, including being dependent on reactive oxygen–nitrogen–sulphur species. One of the key abiotic stresses is drought. As a result of drought, changes in the level of hydration of the plant occur, which obviously entails various metabolic alternations. The primary aim of this study was to determine the relationship between the response of barley to drought and the intensity of stress, therefore investigations were performed under various levels of water saturation deficit (WSD) in leaves at 15%, 30%, and 50%. In barley subjected to drought, most significant changes occurred under a slight dehydration level at 15%. It was observed that the gene expression of 9-*cis*-epoxycarotenoid dioxygenases, enzymes involved in ABA biosynthesis, increased significantly, and led to a higher concentration of ABA. This was most likely the result of an increase in the gene expression and enzyme activity of L-cysteine desulfhydrase, which is responsible for H_2_S synthesis. Our results suggest that the differential water deficit in leaves underlies the activation of an appropriate defence, with ABA metabolism at the centre of these processes. Furthermore, at 15% WSD, a dominant contribution of H_2_O_2_-dependent signalling was noted, but at 30% and 50% WSD, significant NO-dependent signalling occurred.

## 1. Introduction

In response to abiotic stress, plants trigger complex signalling cascades, including synthesis of reactive oxygen species (ROS), reactive nitrogen species (RNS) and reactive sulphur species (RSS) [1,2]. These signalling molecules regulate numerous physiological processes and enable plants to adapt to adverse conditions [3]. ROS, RNS and RSS (headed by hydrogen sulphide, H_2_S) engage in various molecular processes, including the modification of proteins containing thiol groups (-SH), which are particularly vulnerable to oxidation. One of the key elements of antioxidative defence is glutathione. The level of reduced glutathione (GSH), which is involved in the protection of -SH groups against oxidation, increases in plants under abiotic stress [4]. The level of GSH and the activities of the ascorbate-GSH cycle enzymes on the one hand are associated with ROS detoxification, but on the other hand, they are involved in redox signalling under drought stress [5]. Until now, the role of ROS and RNS during plant response against abiotic stress is better discerned than RSS, and thus there is a greater need to research the latter molecules.

One of the roles of H_2_S as a signalling molecule is the post-translational modification of reactive cysteine residues in proteins by their *S*-sulfhydration. H_2_S converts -SH to persulfide bridges (-SSH), leading to functional changes in proteins, which is an analogous mechanism to the *S*-nitrosylation induced by nitric oxide (NO) or other oxidative modifications of cysteine residues, such as *S*-glutathionylation, the meaning of which was described in our previous work [6]. *S*-sulfhydration is an essential element of many basic plant metabolic pathways, such as glycolysis, the tricarboxylic acid cycle, the Calvin cycle, and starch biosynthesis. This modification is the same as *S*-glutathionylation and *S*-nitrosylation can cause changes in the catalytic activity of enzymes, which may induce important physiological effects [7].

There are numerous enzymes in the plant cell that participate in the production of H_2_S. One of the most important is L-cysteine desulfhydrase (DES), whose role is degrading L-cysteine in order to generate H_2_S. It has been shown that H_2_S produced by DES acts upstream of NO to modulate abscisic acid (ABA)-dependent stomatal closure in *Arabidopsis thaliana* [8]. Most studies on *A*. *thaliana* and *Vicia faba* have revealed that H_2_S causes stomata closure by participating in the ABA-induced signalling pathway, which is of particular importance in drought because it prevents tissue dehydration. Moreover, H_2_S is able to induce the production of H_2_O_2_ in stomata indirectly by an activity stimulation of nicotinamide adenine dinucleotide phosphate (NADPH) oxidase producing superoxide free radicals, which also results in stomata closure [9].

It has also been shown that H_2_S induces the expression of *9*-*cis*-*epoxycarotenoid dioxygenase* (*NCED*), which encodes the enzyme responsible for the biosynthesis of ABA [7]. Maintaining an appropriate ABA content in plant tissues is crucial for the response to stresses. Endogenous ABA concentration is the result of balance between simultaneously occurring processes, biosynthesis, and catabolism [10]. The conversion of ABA into an inactive form occurs in a reaction catalysed by ABA 8’-hydroxylase (ABA8’OH) or by the formation of inactive ABA-glucose esters (ABA-GEs), which is catalysed by ABA UDP-glucosyltransferase (UGT) [11].

The main function of the nitrate reductase (NR) is the reduction of NO_3_^−^ to NO_2_^−^. However, under abiotic stress, NR can also reduce NO_2_^−^ to NO, which is a relevant pathway for NO production in higher plants [12]. NO can also react with GSH to form *S*-nitrosoglutathione (GSNO), which is characterized by a broad spectrum of physiological and stress-related activities [13]. GSNO is a natural reservoir of NO in plant cells and can mediate plant stress response throughout specific post-translational modification of redox-sensitive proteins [14] and the regulation of gene expression [15].

The main aim of this study was to analyse the biochemical and molecular parameters related to the response of barley to drought stress. It was assumed that the response of the plant will depend on the intensity of stress; therefore, the study was conducted at various levels of water saturation deficit (WSD) in barley leaves, namely at 15%, 30%, and 50% WSD. To learn more about the molecular basis of barley response to water deficit, we concentrated on the gene expression of enzymes involved in ABA metabolism. In addition, in this article we provide experimental evidence that the gene expression and enzymatic activity of DES, the still poorly understood plant enzyme, alters in leaves of barley plant under drought.

## 2. Results

### 2.1. Response of Barley to Water Deficiency

**Figure 1:**
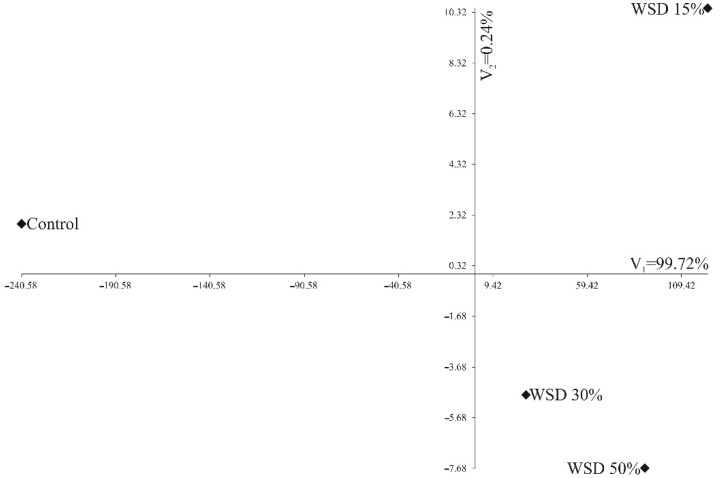
The distribution of water saturation deficit (WSD) variants in the system of the first two canonical variables. A canonical variate analysis (CVA) of the variability of the nine traits of four WSDs in terms of the first two canonical variates was performed.

Water saturation deficit was marked for all the harvested plants. The control plants showed WSD at the level of 8%. Groups of plants treated with drought were harvested at WSD, which reached 15%, 30% and 50%, respectively. Figure 1 shows a canonical variate analysis (CVA) of the variability of the nine traits of four WSDs in terms of the first two canonical variates. In the graph, the coordinates of the point for particular WSDs are the values for the first and second canonical variates, respectively. The first two canonical variates accounted for 99.96% of the total multivariate variability between the individual WSDs (Figure 1).

The greatest variation in terms of all the nine traits jointly measured with Mahalanobis distances was found for the control and 15% WSD (distance between them amounted to 364.1). The greatest similarity was found between 15% WSD and 50% WSD (38.2) (Table 1).

### 2.2. Effect of Dehydration on Lipid Peroxidation, NO_2_^−^, NO_3_^−^, H_2_O_2_ Contents

**Figure 2:**
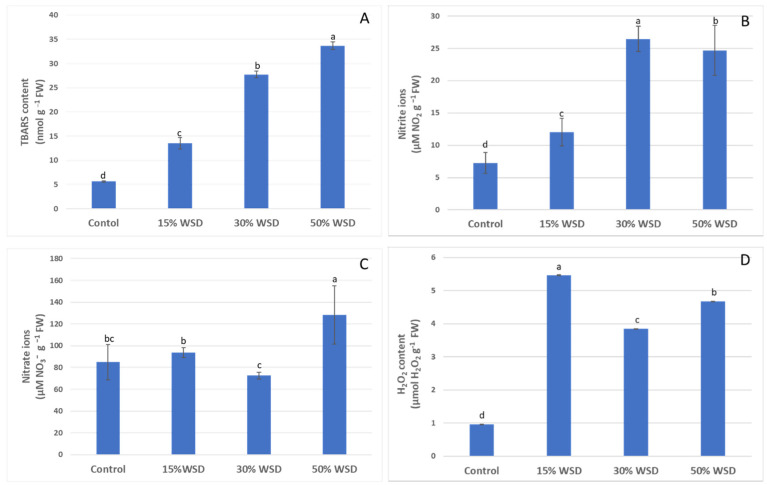
Lipid peroxidation as the 2-thiobarbituric acid reactive substances (TBARS) (**A**), the nitrite ions (NO_2_^−^) (**B**), nitrate ions (NO_3_^−^) (**C**) and the hydrogen peroxide (H_2_O_2_) (**D**) contents in the control and dehydrated to 15%, 30%, and 50% WSD plants. Vertical bars indicate means (*n* = 9) ± standard deviation. Letters indicate Fisher’s least significant differences, which were estimated at a significance level of α = 0.05.

Lipid peroxidation during a water deficit was estimated as the 2-thiobarbituric acid reactive substances (TBARS) content in the well-watered control and plants dehydrated to 15%, 30% and 50% WSD (Figure 2A). The TBARS content increased linearly during dehydration, and it was the highest at the greatest dehydration at 50% WSD of the leaves reaching 33.7 nmol of TBARS per g of fresh weight (FW), which was 6.6 times higher than the lipid peroxidation in control plants.

Similarly, the concentration of NO_2_^−^ increased under dehydration, however it reached the highest value of 26.5 µM NO_2_^−^ per g of FW at 30% of WSD and remained at a comparable level at 50% of WSD (Figure 2B). The content of NO_3_^−^ ions at 15% and 30% of WSD was comparable to the control, although the concentration of these ions was significantly lower at 30% WSD than at 15%. A significant increase in concentration was observed only with deep dehydration at 50% WSD, which led to an increase of 52% of the NO_3_^−^ ions level in comparison to plants grown under control conditions (Figure 2C).

The concentration of H_2_O_2_ rapidly increased under drought stress, and its highest values were recorded at the lowest dehydration level at 15% WSD in the initial phase of dehydration and was over 5.65 times higher than in the control plants. The H_2_O_2_ content remained high during the whole stress period, although the significant drop was noticed at 30% WSD (by 30% in comparison with 15% WSD) (Figure 2D).

### 2.3. Variability of ABA and GSH/GSSG Contents on Dehydration

**Figure 3:**
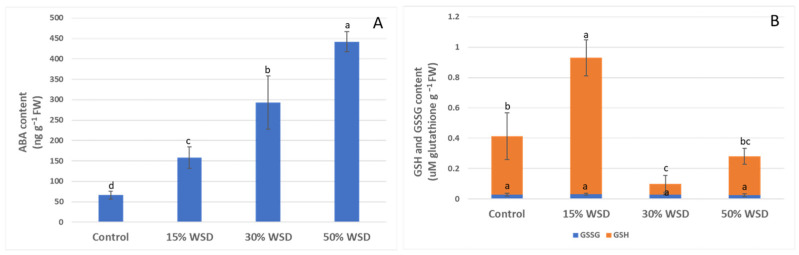
Effect of drought on abscisic acid (ABA) (**A**) and reduced (GSH) and oxidized (GSSG) glutathione (**B**) contents in the control and dehydrated to 15%, 30%, and 50% WSD plants. Vertical bars indicate means (*n* = 9) ± standard deviation. Letters indicate Fisher’s least significant differences, which were estimated at a significance level of α = 0.05.

The level of ABA increased linearly with the intensity of dehydration. At the greatest dehydration at 50% WSD, its level increased more than six times compared to the control plants (Figure 3A). The GSH content in the plant’s response to dehydration was dependent on the WSD level. Reduced glutathione concentration increased by more than double in the first phase of drought stress at 15% WSD, as compared to the control. At 30%, a significant drop in the GSH content was observed. It decreased 12.8-fold in comparison to 15% WSD. At 50% WSD, a GSH content reached a concentration that was comparable with to control plants (Figure 3B). Unlike the reduced form of glutathione, its oxidized form (GSSG) remained constant at all levels of tissue dehydration (Figure 3B).

### 2.4. DES and NR Activities

**Figure 4:**
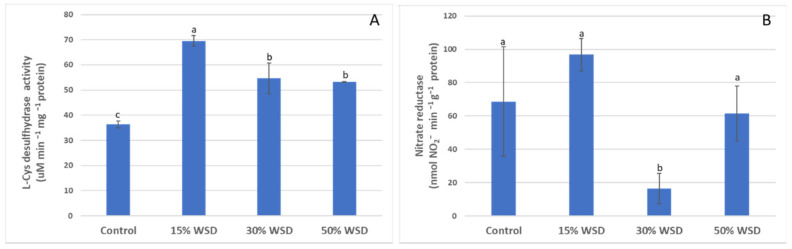
Changes in the L-cysteine desulfhydrase (**A**) and nitrate reductase (**B**) activities in control and dehydrated to 15%, 30%, and 50% WSD plants. Vertical bars indicate means (*n* = 9) ± standard deviation. Letters indicate Fisher’s least significant differences, which were estimated at a significance level of α = 0.05.

The DES activity significantly increased at 15% of WSD by over 90% in comparison to plants grown in optimal conditions. At higher dehydration, the DES activity slightly decreased, however it remained higher by approximately 50% than in the control at both levels of dehydration: 30% and 50% of WSD (Figure 4A). The NR activity was the same as the DES activity that reached the highest value at WSD 15%, and although it was higher by 41% than in the control, the observed difference was not statistically significant. A significant decrease in NR activity was observed at 30% WSD and it was 83% lower than at the initial stage of stress. At 50% WSD, NR activity returned to a level comparable to that of the control plants (Figure 4B).

### 2.5. Analysis of Gene Expression of NCED1, NCED2, ABA8’OH1, ABA8’OH2, UGT1 and DES

**Figure 5:**
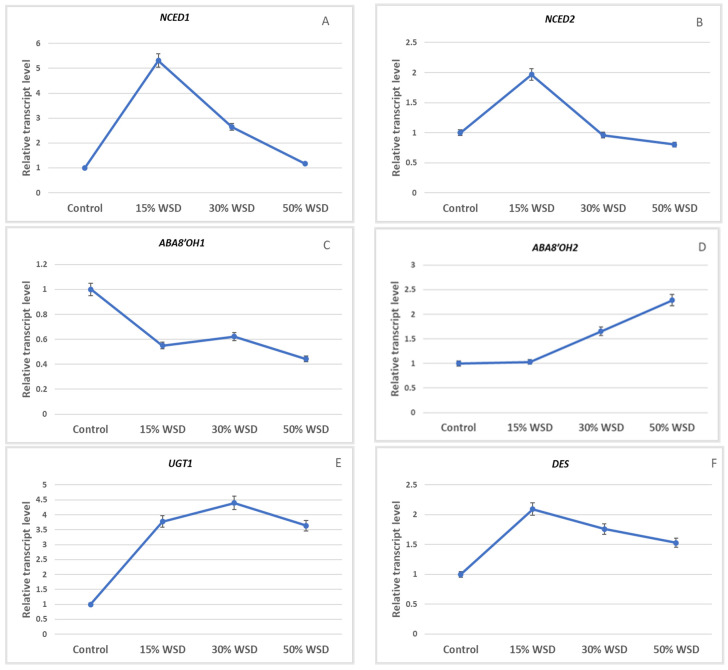
Transcript levels of *NCED1* (**A**), *NCED2* (**B**), *ABA8′OH1* (**C**), *ABA8′OH2* (**D**), *UGT1* (**E**) and *DES* (**F**) in the control and dehydrated plants to 15%, 30%, and 50% WSD. Vertical bars indicate means ± standard deviation of at least three technical replicates of semi-quantitative reverse transcription-PCR performed for each of the two biological replicates.

Expression of genes encoding 9-*cis*-epoxycarotenoid dioxygenase was observed at the highest level in barley leaves at 15% WSD (Figure 5A,B). The increase of transcript levels in comparison to the control was five-fold and two-fold higher for *NCED1* and *NCED2*, respectively. With increasing WSD, a decrease in the expression of these genes was observed. Expression profiles of genes encoding ABA 8’-hydroxylase, responsible for ABA catabolism, were different. The highest level of the *ABA8’OH1* transcript was observed in the control, and a decrease in the expression of this gene was noted in the barley leaves under drought (Figure 5C). In turn, *ABA8’OH2* expression increased in leaves with increasing WSD (Figure 5D). The level of the *UGT1* transcript, encoding the enzyme involved in the formation of ABA-GEs, was highest in leaves at 15% and 30% WSD (Figure 5E). The expression of the gene encoding *DES* was the highest in barley leaves at 15% WSD and an over two-fold increase in the transcript level of this gene was observed, as compared to the control (Figure 5F).

### 2.6. Correlation Analysis

**Figure 6:**
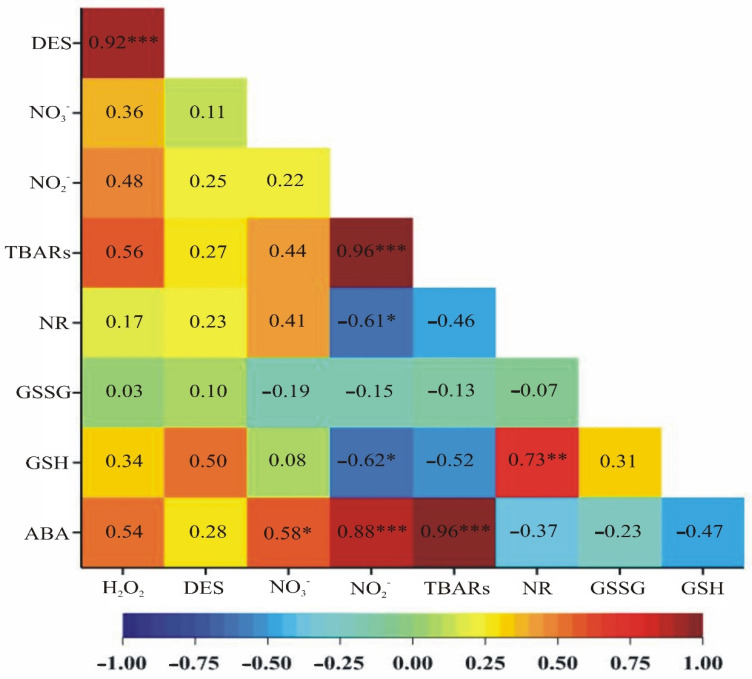
A heat map showing correlation coefficients between ABA, GSH, GSSG, TBARS, H_2_O_2_, NO_3_^−^, NO_2_^−^ contents and DES and NR activities. ***—a significance level of α = 0.001, **—a significance level of α = 0.01, *—a significance level of α = 0.05.

The highest significant positive correlation was observed for ABA content and lipid peroxidation (TBARS) as well as for lipid peroxidation and nitrite ions content (correlation strength 0.96 at a significance level of α = 0.001), following correlation between DES activity and hydrogen peroxide concertation (strength 0.92 at a significance level of α = 0.001) and ABA content and nitrite ions content (strength 0.88 at a significance level of α = 0.001). A slightly lower positive correlation was noticed between reduced glutathione content and nitrate reductase activity (strength 0.73 at a significance level of α = 0.01) and between the ABA content and nitrate ions level (strength 0.58 at a significance level of α = 0.05). Apart from positive correlations, significant negative correlations were also observed. Such correlations were observed for the concentration of nitrite ions and the content of reduced glutathione as well as for the concentration of nitrite ions and the activity of nitrate reductase (strength −0.62 and −0.61, respectively, at a significance level of α = 0.05) (Figure 6).

## 3. Discussion

Adequate water content in plant tissues is necessary for the proper plant growth and development, and in the case of arable crops, to produce high and good-quality crops. Maintaining the optimal water content in tissues requires coordinating plant growth with the water availability for the roots and the rate of water loss in the transpiration process. When this balance is disturbed because of soil drought, acclimatization responses are activated in the plant to adapt to unfavourable conditions, including e.g., closing the stomata, synthesis of compounds that protect cell structures and ensure the maintenance of the integrity of biological membranes [16]. The main research hypothesis of this study is that the varied level of hydration in spring barley leaves translates into contrasting responses that are triggered by water deficit in the plant in response to drought stress. We decided to verify this hypothesis, which we forged based on our previous research on the response of cereals to drought, using molecular and biochemical methods. For our research, we selected parameters that are known to be strongly associated with plant defence responses to diverse types of environmental stresses (abiotic and biotic). We have paid particular attention to the regulation of ABA content in leaves, which is known to be “the hormonal heart” of the plant response to drought stress [17].

A rapid production of ROS, including H_2_O_2_, is one of the earliest activated plant responses against water deficit. Under drought condition, this molecule is among the others synthesized to perform the function of a secondary messenger in ABA-regulated stomata closure [18,19]. While analysing our results, we found several significant correlations between the analysed biochemical parameters. These dependencies indicate a specific phytohormone and signal molecule crosstalk during spring barley’s response to water deficit. This is especially noticeable at 15% WSD. This would be coherent with the regularity that usually, in the first phase (an alarm phase) of the plant stress response (in this particular case 15% WSD), there is an intense liberation of signalling molecules to trigger defence responses against the stress detected by the plant.

It was noticed that, because of the increasing water deficit in barley leaves, and thus the incremental intensity of oxidative stress manifested by an elevated level of TBARS (markers of lipid oxidation), an enhanced content of H_2_O_2_ and ABA and the enzyme activity of DES catabolizing L-cysteine to H_2_S were observed. This specific biochemical triad (H_2_S, H_2_O_2_ and ABA) activated in barley plants struggling with progressive dehydration of leaf tissues may significantly promote the induction and development of drought acclimatization responses dependent on these three signalling molecules. It is highly likely that the end physiological effect is the closure of the stomata by barley plants, which reduces water loss through transpiration, thus maintaining a relatively optimal water level in the leaves is possible and water stressed barley plants can more effectively deal with the progressive secondary stress, namely oxidative stress. This claim is confirmed by numerous studies showing that H_2_S induced stomata closure by mediating in the ABA-dependent signalling, and what is especially important is that H_2_S stimulated the synthesis of H_2_O_2_ in stomata, which led to stomata closure as well [7]. It has also been shown that the level of glutathione (which is part of the non-enzymatic antioxidant machinery) and its dependent responses to abiotic stresses are regulated by H_2_S [20,21]. We noted that GSH concentration increased more than double in the first phase of drought stress at 15% WSD compared to the control plants, thus suggesting its beneficial effect on the effectiveness of maintaining the redox balance in barley leaf cells at this stage of the response to drought.

As already mentioned above, ABA is a regulatory hub of phytohormone and other signalling molecule levels during plant water deficit stress responses, thus we took a closer look at the expression level of genes that participate in ABA metabolism. Many of the mechanisms of plant response to water scarcity depend on the ABA content in plant organs. In the tissues of plants undergoing drought, a several, or even several dozen, increase in the content of this phytohormone is observed [22]. Indeed, in barley leaves, the ABA content increased linearly with the exacerbation of dehydration, which allowed for the continued functioning of plants that were exposed to the lack of water.

Until recently, it was postulated that soil drought stimulates the biosynthesis of ABA in the roots, which is then transported through the xylem to shoots and leaves, where it reaches the guard cells and induces the closure of the stomata [23]. However, as studies have shown, when Arabidopsis roots and shoots were subjected to water deficit separately, an increase in ABA content was only observed in the shoots, indicating that they are the main sites for ABA synthesis in response to water deficit [24]. We observed that, in the leaves of barley plants subjected to drought, increased ABA biosynthesis takes place, which was indicated by an increased level of transcripts of genes encoding 9-*cis*-epoxycarotenoid dioxygenase—NCED1 and NCED2. It can be assumed that NCED1, whose increase in gene expression was higher in stressed plants at 15% WSD compared to the control, than that of NCED2, played a more significant role in the accumulation of ABA in the leaves of barley plants growing in the conditions of water deficit. Next, with increasing WSD, a decrease in the expression of these genes occurred. Similarly, in wheat leaves, dehydration caused a significant increase in the transcript level of one of the genes encoding NCED—*TaNCED1* [25]. In turn, among the five genes encoding NCED in the Arabidopsis, expression of only one, *AtNCED3*, was strongly induced by drought, and overexpression of this gene resulted in increased ABA accumulation and increased drought tolerance [26]. Thus, in both cereals and model plants, the water deficit induced the expression of genes encoding NCED, hence this enzyme plays a key role in the regulation of ABA biosynthesis in response to drought.

The physiological effects mediated by ABA require the maintenance of an appropriate content of this phytohormone in plant tissues. Hence, in plants subjected to various stress factors, often intensive ABA biosynthesis, it is accompanied by an increase in the transcript level of genes encoding enzymes involved in ABA catabolism, wherein conversion of the ABA to an inactive form takes place due to ABA8’OH activity [27]. The genes encoding this enzyme belong to the cytochrome P450 family [28]. Four genes encoding ABA8’OH were found in the Arabidopsis. These genes showed tissue-specific patterns of expression that were altered by various environmental stimuli [27]. It has been shown that the transcript level of genes encoding ABA8’-OH increased in leaves of dehydrated Arabidopsis plants [29]. In turn, the *ABA8*’*OH2* expression increased in leaves of barley plants with progressive water shortage, suggesting that ABA8’OH2 plays an essential role in the breakdown of ABA molecules during barley response to drought.

In leaves of barley plants growing under drought at 15% and 30% WSD, intensive ABA biosynthesis seems to coexist with the formation of inactive ABA-GEs, as evidenced by the increased level of the UGT1 transcript compared to control plants. ABA-GEs can be a transport form of this phytohormone and can also be stored in plant tissues [22,30]. ABA-GE are hydrolysed by β-glucosidase, resulting in the liberation of biologically active ABA molecules [31]. The hydrolysis of ABA-GE to ABA occurs in one step and compared to de novo ABA biosynthesis, it can lead to an increase in the content of this phytohormone in plant tissues much faster [32]. ABA-GEs formation and deconjugation may therefore play a vital role in increasing local ABA concentration in response to stress factors. The elevated level of the UGT1 transcript in the leaves of barley plants subjected to drought may indicate the accumulation of ABA in a conjugated form, which is a reserve of this phytohormone in the event of a deepening water deficit, which can be quickly activated in the process of deconjugation. Similarly, an increase in the transcript level of the UGT-encoding gene was observed in drought-treated *Vigna angularis* [33].

Plants need nitrogen (N) for developmental processes and defence against abiotic stress. A series of reactions catalysing by oxidoreductases (NO_3_^−^→NO_2_^−^→NH_4_^+^), enabling N assimilation and biosynthesis of amino acids by transamination, is one of the main points of regulation of defence responses of plants against stresses [34]. The first of these reactions is catalysed by NR. We would like to bring to attention here that NO_3_^−^ conversion to NO_2_^−^ by NR is a restrictive step in N assimilation [35]. Thus, increased NR activity in barley leaves at 15% WSD, although the observed difference was not statistically significant in comparison with control plants, may promote the de novo synthesis of nutrients. Considering the participation of NR in response to abiotic stress, it is impossible not to emphasis its other physiological role, namely the ability to synthesise NO. We only mention this and hypothesize that the synthesis of NO by NR may take place, but we have no direct experimental evidence for it, so this issue requires further investigation soon.

Another interesting aspect related to NO metabolism is the observed coincidence revealed by statistical analysis and showing a significant positive correlation (strength 0.96 at a significance level of α = 0.001) for lipid peroxidation and nitrites, major end products of NO turnover. In addition, in barley leaves at 30% and 50% WSD, this trend was accompanied by a reduced level of H_2_O_2_ compared to samples collected at 15% WSD. Thinking about the interpretation of these results, we conducted experiments to describe equivalent results in the field of vertebrate biochemistry. Van der Vliet et al. [36] revealed that NO_2_^−^ can be oxidized by the haem peroxidases, such as horseradish peroxidase, myeloperoxidase (MPO), and lactoperoxidase (LPO), in the presence of H_2_O_2_, to nitrogen dioxide radical, which can promote tyrosine nitration. These authors concluded that NO_2_**^−^**, both at physiological and pathological contents, can be a substrate for the mammalian MPO and LPO and that production of nitrogen dioxide radicals by peroxidase-catalysed oxidation of nitrites can be a supplementary pathway inducing cytotoxicity or defence response associated with NO signalling. However, Byun et al. [37] showed that nitrogen dioxide radical produced by the MPO-H_2_O_2_^−^ NO_2_^−^ system was responsible for lipid peroxidation of low-density lipoprotein. We cannot rule out that this phenomenon, known from research on animal models, has been detected by us in our plant model. This is supported by the fact that the horseradish peroxidase mentioned above is also capable of oxidizing NO_2_^−^ to nitrogen dioxide radical in the presence of H_2_O_2_. We have already undertaken research efforts to verify whether this mechanism actually works effectively in plants.

## 4. Materials and Methods

### 4.1. Plant Material

All experiments were conducted on spring barley (*Hordeum vulgare* L.) ‘Airway’. It is a variety of fodder barley with high prolificacy and protein content in kernels. It is recommended for medium-intensive and intensive cultivation technology on moderate-quality soils. Spring barley ‘Airway’ is highly resistant to *Rhynchosporium secalis*, *Drechslera sorokiniana* and *Blumeria graminis* f. sp. *hordei* and good to *Pyrenophora teres* and *Puccinia hordei*. Plants are of medium height with particularly good resistance to lodging. The advantage of this variety is high resistance to peduncle breakage.

Initially, grains were soaked in water for 2 h and then surface decontaminated in 5% sodium hypochlorite solution. Then, in order to eliminate microbial contamination, the grains were incubated for one hour on a magnetic stirrer in 0.2% Plant Preservative Mixture™ (PPM) (Plant Cell Technologies, Inc., Washington, DC, USA). The decon-taminated grains were placed in a petri dish on filter paper soaked in 0.2% PPM. After a two-day incubation of the grains at 23 °C in the darkness, they were transferred to pots (four grains per pot) filled with commercial potting mix. Plants were grown in a growth chamber at a temperature of 23 ± 2 °C and 18 °C with a 16 h day (100 ± 25 µmol m^−2^ s^−1^ light)/8 h night photoperiod, respectively, and under 70–80% humidity.

After fourteen days of growth under optimal conditions, drought was induced by the cessation of water supply to the pots. The research material consisted of plants growing under control conditions and subjected to drought stress at various stress levels at 15, 30% and 50% WSD. To put it in detail, after the cessation of irrigation, the plants were incubated in the growth chamber for a further six days, during which time the WSD in the leaves reached 15%, after another three days 30% and after another two days 50%. Appropriate control plants were sampled. All plants (control and stressed) were in the same stage of development, namely at the beginning of tillering.

### 4.2. Measurement of WSD

The dehydration of spring barley leaves was determined as the percentage of WSD, calculated according to Turner [38], as follows:

$$\mathrm{WSD}=\frac{\mathrm{Water}\;\mathrm{saturated}\;\mathrm{mass}-\mathrm{actual}\;\mathrm{fresh}\;\mathrm{mass}}{\mathrm{Water}\;\mathrm{saturated}\;\mathrm{mass}-\mathrm{dry}\;\mathrm{mass}}\times 100\%$$

Water saturated mass was measured after submersion of leaves overnight in water in the dark, and the dry mass was determined after drying the tissue at 80 °C for 16 h. Actual fresh mass weight was measured right after sample collection.

### 4.3. L-Cysteine Desulfhydrase (DES) Activity

The activity of DES was determined using a modified method of Alvarez et al. [39]. 100 mg of leaf tissue was homogenized in liquid nitrogen and 1 mL of 20 mM Tris-HCl buffer (pH 8.0) was added. The homogenate was centrifuged for 15 min at 13,200× g at 4 °C and the supernatant was collected. To 10 µL of the supernatant the reaction mixture which consisted of 1 mM dithiothreitol (DTT), 1 mM L-Cysteine (L-Cys) in 100 mM Tris-HCl buffer (pH 8.0) was added to make a final volume of 100 µL. After 15 min of incubation at 37 °C, the enzymatic reaction was stopped by adding 10 µL of 30 mM iron (III) chloride dissolved in 1.2 M HCl and 10 µL of 20 mM N,N-dimethyl-p-phenylalanine dihydrochloride (DMPPDA) dissolved in 7.2 M HCl. The DES activity was measured photometrically by observing the changes in absorbance at 670 nm. Measurements were made in Nunc U-bottom 96-well plate (Thermo Scientific, Waltham, MA, USA) using a Varioskan LUX Multimode Microplate Reader (Thermo Scientific, Waltham, MA, USA). The enzyme activity was expressed in µM methylene blue (MB) min^−1^ mg^−1^ protein. Extinction coefficient for MB is 3.1 mM^−1^ cm^−1^ was used for the calculations. Protein content was measured according to the Bradford [40] method.

### 4.4. NO_3_^−^ and NO_2_^−^ Content

Determination of the nitrate and nitrite content was made with the Nitric Oxide Assay Kit (Invitrogen/Thermo Scientific, Waltham, MA, USA) according to the manufacturer’s instructions. The levels of nitrates and nitrites were determined photometrically at the wavelength of 540 nm. Measurements were made in Nunc U-bottom 96-well plate (Thermo Scientific, Waltham, MA, USA) using a Varioskan LUX Multimode Microplate Reader (Thermo Scientific, Waltham, MA, USA). To determine the results, standard curves made with the use of standards available in the kit were used. The final results were expressed in µM of NO_3_^−^ and NO_2_^−^ per gram of FW.

### 4.5. Lipid Peroxidation

Lipid peroxidation was measured by the Heath and Packer [41] method. 100 mg of leaves were homogenized in 0.5 mL of 0.1% (*w*/*v*) trichloroacetic acid (TCA). The homogenate was centrifuged for 10 min at 13,200× *g* at 4 °C and the supernatant was collected. 1.5 mL of 0.5% (*w*/*v*) 2-thiobarbituric acid dissolved in 20% TCA was added to 0.5 mL of the supernatant. The mixture was incubated at 95 °C for 25 min and the reaction was stopped by transferring samples on ice. After cooling, 200 µL of each mixture was taken and pipetted into Nunc U-bottom 96-well plate (Thermo Scientific, Waltham, MA, USA). The absorbance of the supernatant was measured at 532 nm, with a reading at 600 nm subtracted (nonspecific turbidity) using a Varioskan LUX Multimode Microplate Reader (Thermo Scientific, Waltham, MA, USA). The TBARS content was determined using an extinction coefficient of 155 mM^−1^ cm^−1^ (to quantify lipid peroxides). It was expressed as nmol of TBARs per gram of FW.

### 4.6. Nitrate Reductase (NR) Activity

The activity of nitrate reductase was determined by the modified Jaworski method [42]. 100 mg of the leaves were homogenized in liquid nitrogen and 1 mL of 0.1 M Tris-HCl buffer (pH 7.5) was added. The homogenate was centrifuged for 15 min at 13,200× *g* at 4 °C and the supernatant was collected. To 45 µL of the reaction mixture which consisted of 5% (*v*/*v*) propanol and 30 mM KNO_3_ dissolved in 0.1 M phosphate buffer (pH 7.5) and 5 µL of supernatant was added. The mixture was incubated for 30 min at 30 °C and 50 µL of a solution consisting of 1% (*w*/*v*) sulphanilamide and 0.02% (*v*/*v*) N-(1-naphthyl)ethylenediamine hydrochloride in 3 M HCl was added. The absorbance at 540 nm was measured in Nunc U-bottom 96-well plate (Thermo Scientific, Waltham, MA, USA) on a Varioskan LUX Multimode Microplate Reader (Thermo Scientific, Waltham, MA, USA). Protein content was determined by the Bradford [40] method. The NR activity was calculated using a standard curve and expressed in nM NO_2_^−^ per min per mg of protein.

### 4.7. Glutathione Content

Reduced glutathione was assayed following the method described by Gronwald et al. [43]. 200 mg of leaves was ground to a powder in liquid nitrogen and then homogenized in 7.57 mM sodium ascorbate and incubated for 30 min at 4 °C. Next, the homogenate was centrifuged for 15 min at 16,000× *g* at 4 °C. The supernatant was used for the total glutathione (GSH + GSSG) assay and for the oxidized glutathione (GSSG) assay. N-ethylmaleimide (NEM), which masks thiol groups in GSH, was used to determine GSSG. 1 mL of the reaction mixture consisted of 0.4 mL of extract and 10 mM NEM dissolved in 125 mM sodium-phosphate buffer (pH 7.5) containing 5 mM EDTA. The mixture was incubated at room temperature for 70 min and then NEM was removed by extraction with ether, which was repeated 5 times. Glutathione was determined as described by Ellman [44] with the 5,5′-dithiobis (2-nitrobenzoic acid) (DTNB). The reaction mixture for the assay of GSSG or total glutathione consisted of 10 mM phosphate buffer (pH 7.0), 0.5 mM NADPH, 0.12 units glutathione reductase and supernatant. An increase in the absorbance at 412 nm was measured after the addition of DTNB. The total glutathione (GSH + GSSG) and GSSG contents were calculated using a standard curve and expressed in the µM of glutathione per gram of FW.

### 4.8. Determination of Endogenous ABA Content

The endogenous ABA concentration was measured using the Phytodetek ABA enzyme immunoassay test kit (Agdia, Inc., Elkhart, IN, USA). A sample of 250 mg of frozen barley leaves was homogenized in liquid nitrogen, then 1.5 mL of extraction buffer (80% methanol, 2% acetic acid and 20 mg/l butylated hydroxytoluene) was added to the powdered tissue. The ABA was extracted and described by Fidler et al. (2018) [45]. The ABA content was measured in triplicate.

### 4.9. Total RNA Extraction

Total RNA was isolated from the barley leaves using the GeneMATRIX Universal RNA Purification Kit (EURx, Gdańsk, Poland), including the RNase-free DNase I (EURx, Gdańsk, Poland) treatment according to the manufacturer protocol.

### 4.10. Semi-Quantitative Reverse Transcription-PCR (sqRT-PCR)

The transcript levels of the genes encoding enzymes engaged in ABA metabolism and DES in barley leaves were determined using Titanium One-Step RT-PCR Kit (Clontech Laboratories, Mountain View, CA, USA). sqRT-PCRs were performed with gene-specific primers and aliquots of 100 ng of total RNA were used as templates in each reaction. As an internal control amplification of the 18S rRNA gene was performed. The reactions were performer under following conditions: 60 min at 50 °C; 5 min at 94 °C; 8 (*18S rRNA*), 36 (*NCEDs*), 35 (*ABA8′OH1*, *DES*), 32 (*ABA8′OH2*), or 33 (*UGT1*) cycles of 30 s at 94 °C, 30 s at 61 °C (*NCEDs*), 60 °C (*ABA8′OH1, UGT1*), or 62 °C (*18S rRNA*, *ABA8′OH2*, *DES*), 30 s at 68 °C; and a final extension of 5 min at 68 °C. Relative transcript levels were quantified using the software ImageJ 1.53m version (U. S. National Institutes of Health, Bethesda, MD, USA). Representative images of gel and primer sequences are provided in Figure S1 and Table S1 in Supplementary Materials.

### 4.11. Statistical Analysis

Three independent experiments in three replicates (*n* = 9) were performed. The normality of the distribution of the nine traits, i.e., H_2_O_2_, DES, NO_3_^−^, NO_2_^−^, TBARS, NR, GSH, GSSG and ABA, was verified with Shapiro–Wilk’s normality test to check whether the analysis of variance (ANOVA) met the assumption that the ANOVA model residuals followed normal distribution. The homogeneity of variance was evaluated using Bartlett’s test. Box’s M test was used to check multivariate normality and homogeneity of variance-covariance matrices. All the traits had normal distribution. A one-way (WSD) multivariate analysis of variance (MANOVA) was performed. Following this, one-way analyses of variance (ANOVA) were performed to verify the null hypotheses of a lack of WSD effect on the nine observed traits, independently for each one. The arithmetic means, minimal and maximal values as well as standard deviations were calculated. Moreover, Fisher’s least significant differences (LSDs) were estimated at a significance level of α = 0.05. The relationships between observed traits were estimated using Pearson’s linear correlation coefficients. The results were also analysed using multivariate methods. A canonical variate analysis was applied to present a multi-trait assessment of the similarity of the tested WSDs in a lower number of dimensions with the least possible loss of information. The Mahalanobis distance was suggested as a measure of “polytrait” WSDs similarity [46], the significance of which was verified by means of critical value Dα called “the least significant distance” [47]. The Mahalanobis distances were calculated for all pairs of WSDs. The GenStat v. 18 statistical software package (VSN International) was used for the analyses. All statistical analyses are presented in Table S2 in Supplementary Materials.

## 5. Conclusions

In conclusion, our study indicates that the specific responses of spring barley plants against drought are related to the intensity of water stress *in planta* measured as WSD in leaves. In barley plants exposed to drought, the most noticeable molecular and metabolic changes took place under slight dehydration level at 15% WSD. It was noted that the gene expression of 9-*cis*-epoxycarotenoid dioxygenases, enzymes involved in ABA biosynthesis, was significantly enhanced, which led to an elevated level of ABA. This was accompanied by an increase in the gene expression and enzyme activity of DES, which is responsible for H_2_S production. These discoveries are consistent with the outlook that during the first phase (an alarm phase) of the plant ecophysiological reaction (in our experimental case at 15% WSD) there is an intense biosynthesis of signalling molecules to trigger defence against stressful environmental conditions. Furthermore, results indicate a dominant contribution of H_2_O_2_ signalling at 15% WSD, while at 30% and 50% WSD a significant role is played by NO signalling as increased level of nitrites (major end products of NO metabolism) was noted.

## Figures and Tables

**Table 1:** The Mahalanobis distances between water saturation deficit (WSD) levels based on all nine traits.

WSD	Control	15% WSD	30% WSD	50% WSD
Control	0			
15% WSD	364.1	0		
30% WSD	267.8	97.7	0	
50% WSD	330.8	38.2	63.6	0

## Data Availability

Not applicable.

## Supplementary Materials

The following supporting information can be downloaded at: https://www.mdpi.com/article/10.3390/ijms232315240/s1.
