# Supplementary material for: Differential Water Deficit in Leaves Is a Principal Factor Modifying Barley Response to Drought Stress
Source: Int J Mol Sci. 2022 Dec 3;23(23):15240. doi: 10.3390/ijms232315240 (PMC9739961; doi:10.3390/ijms232315240)
Supplement: Supplementary file 1 [file ijms-23-15240-s001.zip › Table S1.docx]

| Gene | Forward primer | Reverese primer |
| --- | --- | --- |
| *18S rRNA* | CCAGGTCCAGACATAGTAAG | GTACAAAGGGCAGGGACGTA |
| *NCED1* | CTCCATCTTCAACGACACGGACGACC | CAGGTGTGGTAAATGAACCAAGGAATCG |
| *NCED2* | GTGGAGAGGCAGGAGAAGAAGCTC | CTGCTGCAGGCGCTCCGTCTC |
| *ABA8′OH1* | CTTCGGGGAGGAGGAGATGCAG | GTTGTCGCCGAGGAACTTGACCATC |
| *ABA8′OH2* | GATGGCCTTCTTCCTCCTCCTGTGCATC | GTAGTCGCCCTGGTGGAAGAAGAGC |
| *UGT1* | CTGTGCAGGTGTGCCCATGATCAC | GCATTCGTCTTGTTTCCCATTTCTTG |
| *DES* | CAACGGCACCTTCGGCTGCTGC | CAGAAGAACCACTTGTGGAGGTTGCTG |

**Supplementary Table S1.** Gene-specific primers (sequence 5′ → 3′) used for expression analysis
