# Supplementary material for: Differential Water Deficit in Leaves Is a Principal Factor Modifying Barley Response to Drought Stress
Source: Int J Mol Sci. 2022 Dec 3;23(23):15240. doi: 10.3390/ijms232315240 (PMC9739961; doi:10.3390/ijms232315240)
Supplement: Supplementary file 1 [file ijms-23-15240-s001.zip › Table S2.docx]

**Table S2.** Statistical analyses of the H_2_O_2_, NO_3_^-^, NO_2_^-^, 2-thiobarbituric acid reactive substances (TBARS), oxidized glutathione (GSSG), reduced glutathione (GSH), abscisic acid (ABA) contents and enzyme activities of L-cysteine desulfhydrase (DES) and nitrate reductase (NR). Different letters indicate means that are significantly different. ANOVA - analysis of variance, LSD - least significant difference, ns - not significant, SD - standard deviation, WSD - water saturation deficit. *** - a significance level of α=0.001, ** - a significance level of α=0.01.

H_2_O_2_

| WSD | Mean | Min-Max | SD |
| --- | --- | --- | --- |
| Control | 0.967 d | 0.96-0.974 | 0.00706 |
| WSD 15% | 5.464 a | 5.434-5.494 | 0.0297 |
| WSD 30% | 3.843 c | 3.836-3.849 | 0.0065 |
| WSD 50% | 4.672 b | 4.654-4.69 | 0.01776 |
| LSD_0.05_ | 0.034 |  |  |
| *F*-ANOVA | 35808*** |  |  |

NO_2_^-^

| WSD | Mean | Min-Max | SD |
| --- | --- | --- | --- |
| Control | 7.24 d | 7.21-7.26 | 0.0271 |
| WSD 15% | 12.03 c | 11.19-12.92 | 0.8668 |
| WSD 30% | 26.47 a | 26.2-26.74 | 0.2677 |
| WSD 50% | 24.67 b | 23.44-25.91 | 1.2346 |
| LSD_0.05_ | 1.44 |  |  |
| *F*-ANOVA | 455.18*** |  |  |

TBARs

| WSD | Mean | Min-Max | SD |
| --- | --- | --- | --- |
| Control | 5.65 d | 5.49-5.73 | 0.1432 |
| WSD 15% | 13.55 c | 12.14-14.29 | 1.2221 |
| WSD 30% | 27.76 b | 26.94-28.19 | 0.7076 |
| WSD 50% | 33.7 a | 32.84-34.35 | 0.7738 |
| LSD_0.05_ | 1.52 |  |  |
| *F*-ANOVA | 757.99*** |  |  |

GSSG

| WSD | Mean | Min-Max | SD |
| --- | --- | --- | --- |
| Control | 0.02889 a | 0.01846-0.03686 | 0.009441 |
| WSD 15% | 0.03175 a | 0.02458-0.03757 | 0.006597 |
| WSD 30% | 0.02895 a | 0.02594-0.0305 | 0.002603 |
| WSD 50% | 0.02587 a | 0.01151-0.04556 | 0.017641 |
| LSD_0.05_ | 0.01998 |  |  |
| *F*-ANOVA | 0.15 ns |  |  |

GSH

| WSD | Mean | Min-Max | SD |
| --- | --- | --- | --- |
| Control | 0.3851 b | 0.2629-0.5596 | 0.15515 |
| WSD 15% | 0.8976 a | 0.7945-1.0251 | 0.11724 |
| WSD 30% | 0.0706 c | 0.0155-0.1244 | 0.05448 |
| WSD 50% | 0.2556 bc | 0.1992-0.3033 | 0.05262 |
| LSD_0.05_ | 0.1965 |  |  |
| *F*-ANOVA | 34.64*** |  |  |

ABA

| WSD | Mean | Min-Max | SD |
| --- | --- | --- | --- |
| Control | 66.5 d | 55.7-73.7 | 9.54 |
| WSD 15% | 157.9 c | 140.9-188.3 | 26.34 |
| WSD 30% | 293.3 b | 218-336.6 | 65.44 |
| WSD 50% | 441.8 a | 413.5-458 | 24.64 |
| LSD_0.05_ | 70.9 |  |  |
| *F*-ANOVA | 56.69*** |  |  |

DES

| WSD | Mean | Min-Max | SD |
| --- | --- | --- | --- |
| Control | 36.33 c | 35.49-37.93 | 1.382 |
| WSD 15% | 69.56 a | 67.22-71.44 | 2.148 |
| WSD 30% | 54.61 b | 47.79-59.56 | 6.104 |
| WSD 50% | 53.22 b | 53.09-53.35 | 0.131 |
| LSD_0.05_ | 6.23 |  |  |
| *F*-ANOVA | 50.6*** |  |  |

NR

| WSD | Mean | Min-Max | SD |
| --- | --- | --- | --- |
| Control | 68.48 a | 35.58-101.38 | 32.9 |
| WSD 15% | 96.76 a | 87.03-106.48 | 9.72 |
| WSD 30% | 16.47 b | 7.44-25.49 | 9.02 |
| WSD 50% | 61.52 a | 45.08-77.96 | 16.44 |
| LSD_0.05_ | 36.81 |  |  |
| *F*-ANOVA | 8.68** |  |  |
