# Supplementary figures and images for: Differential Water Deficit in Leaves Is a Principal Factor Modifying Barley Response to Drought Stress
Source: Int J Mol Sci. 2022 Dec 3;23(23):15240. doi: 10.3390/ijms232315240 (PMC9739961; doi:10.3390/ijms232315240)

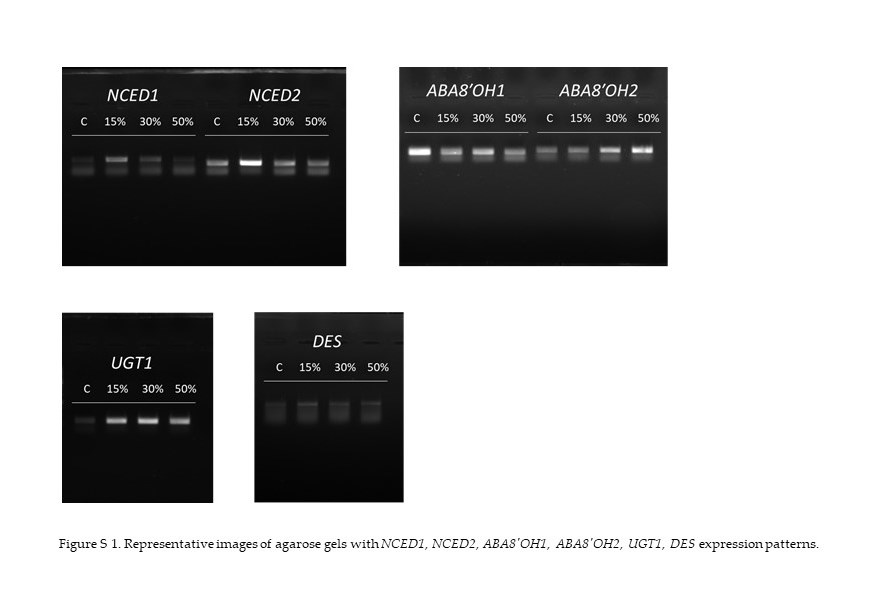

Supplement: Supplementary file 1 [file ijms-23-15240-s001.zip › Figure S1.jpg]
